# Decreased ubiquitin modifying enzyme A20 associated with hyper-responsiveness to ovalbumin challenge following intrauterine growth restriction

**DOI:** 10.1186/s12931-023-02360-2

**Published:** 2023-02-14

**Authors:** Xuefeng Xu, Fei Zheng, Shanshan Xu, Minfei Hu, Chengcheng Hang, Lingke Liu, Chencong Shen, Weizhong Gu, Lizhong Du

**Affiliations:** 1 Department of Rheumatology Immunology & Allergy, Children’s Hospital, Zhejiang University School of Medicine, National Clinical Research Center for Child Health, Hangzhou, 310052 People’s Republic of China; 2 Department of Pathology, Children’s Hospital, Zhejiang University School of Medicine, National Clinical Research Center for Child Health, Hangzhou, 310052 People’s Republic of China; 3 Department of Neonatology, Children’s Hospital, Zhejiang University School of Medicine, National Clinical Research Center for Child Health, Hangzhou, 310052 People’s Republic of China

**Keywords:** A20, Asthma, Intrauterine growth restriction, RNA modification, Ubiquitination

## Abstract

**Background:**

Intrauterine growth restriction (IUGR) is strongly correlated with an increased risk of asthma later in life. Farm dust protects mice from developing house dust mite-induced asthma, and loss of ubiquitin modifying enzyme A20 in lung epithelium would abolish this protective effect. However, the mechanisms of A20 in the development of asthma following IUGR remains unknown.

**Methods:**

An IUGR rat model induced by maternal nutrient restriction was used for investigating the role of A20 in the response characteristics of IUGR rats to ovalbumin (OVA) challenge. The ubiquitination of proteins and N6-methyladenosine (m6A) modifications were used to further assess the potential mechanism of A20.

**Results:**

IUGR can reduce the expression of A20 protein in lung tissue of newborn rats and continue until 10 weeks after birth. OVA challenging can increase the expression of A20 protein in lung tissue of IUGR rats, but its level was still significantly lower than the control OVA group. The differentially ubiquitinated proteins in lung tissues were also observed in IUGR and normal newborn rats. Furthermore, this ubiquitination phenomenon continued from the newborn to adulthood. In the detected RNA methylations, m6A abundance of the motif GGACA was the highest. The higher abundances of m6A modification of A20 mRNA from IUGR were negatively correlated with the trend of A20 protein levels.

**Conclusion:**

These findings indicate A20 as a key regulator during the development of asthma following IUGR, providing further insight into the prevention of asthma induced by environmental factors.

## Introduction

The epidemiological evidence showed that intrauterine growth restriction (IUGR) or lower birth weight is strongly correlated with an increased risk of adult diseases, such as type 2 diabetes mellitus, hypertension, and cardiovascular disease. This is called “developmental origins of adult disease” theory or the “Barker” hypothesis. Recently, more and more studies have focused on the effects of IUGR on later respiratory diseases. There is evidence that IUGR impacts on conducting airways function of very preterm infants at school age, without ventilation inhomogeneity and diffusing capacity [1]. Compared with preterm delivery with appropriate for gestational age, preterm delivery with fetal growth restriction presented with increased susceptibility to asthma and atopic dermatitis [2]. Our meta-analysis also revealed that low birth weight significantly increases the risk of childhood asthma [3]. Furthermore, there exists a significant negative association between birth weight and the development of asthma [4]. These studies demonstrated that children born with IUGR or low birth weight have a significantly increased risk of asthma later in life. However, the underlying mechanism is not fully understood.

In addition to epigenetic regulation and microbiota, more attention has been paid to the effect of ubiquitin-modifying enzyme A20 on the association between an early life environment and asthma later in life [5–7]. A20, also called tumor necrosis factor α-induced protein 3 (TNFAIP3), is a negative regulator of the inflammatory NF-κB pathway [8]. A large number of studies have confirmed that A20 is important for immune regulation in asthma [9, 10]. Chronic exposure to low-dose endotoxin or farm dust protects mice from developing house dust mite-induced asthma, while loss of A20 in lung epithelium would abolish this protective effect, indicating A20 as a key regulator during childhood asthma development and its environmentally mediated protection [8].

Our previous study showed that the maternal nutrient restriction increased the histone acetylation levels in the endothelin 1 (ET1) gene promoter of lung tissue in the newborn rat, continued until 10 weeks after birth. Furthermore, these epigenetic changes induced IUGR individual later in life to be highly sensitive to allergen challenge, resulting in more significant asthma pathological changes [11]. Based on the above studies, we speculated that IUGR might decrease the expression of A20 of lung tissue in the newborn rat and continue later in life, leading to more significant asthma phenotype. The decreased A20 could be associated with RNA methylation. Given the ubiquitination effect of A20, we also hypothesized that there would be an abnormal ubiquitination of protein in lung tissue of IUGR rats. In the present study, an IUGR rat model was used for investigating the role of A20 in the response characteristics of IUGR rats to allergen challenge, and its potential mechanism.

## Materials and methods

### IUGR rat model

This study was carried out in strict accordance with the recommendations in the Guide for the Care and Use of Laboratory Animals of the National Institutes of Health. All procedures and protocols were approved by the Committee on the Ethics of Animal Experiments of Zhejiang University. The IUGR rat model was established based on our previous study [11, 12]. Virgin female SD rats weighing 250–300 g were mated overnight. The pregnant rats were randomly divided into two nutritional groups: standard diet throughout gestation (Control group), and undernutrition group (fed at 50% standard diet). Both groups of rats were kept in the same room and free to drink. Those pups whose birth weight was below the 10th percentile of normal birth weight were defined as IUGR rats. Newborn IUGR rats continued to be reared by diet-restricted mothers that receive normal food intake through lactation, the control pups were reared by control mothers. Both group rats were raised until 6 weeks of age.

### Sensitization and ovalbumin challenge model

Six-week IUGR and control rats were respectively sensitized by intraperitoneal injection of 100 mg of ovalbumin (OVA, Sigma) together with 100 mg Al (OH)_3_ in 1 ml 0.9% NaCl on the 1st day of the 7th and 8th weeks. After two-week sensitization, both groups were exposed to the aerosolized OVA for two consecutive weeks. The aerosol was generated with an ultrasonic nebulizer and was drawn into an exposure chamber containing awake animals. The concentration of OVA in the nebulizer was 10%, and the duration of exposure was 30 min. Rats were sacrificed 12 h after final OVA challenge, and lung tissues were removed, frozen in liquid nitrogen and stored at − 80 °C for further study.

### Western blot detection

Total protein extracts and protein concentrations were prepared according to our previous studies [11, 13]. In brief, total protein extracts were prepared from lung tissues in a RIPA lysis buffer containing protease inhibitors. Protein concentrations were measured using a BCA assay method. Thirty microgram protein extracts were resolved on 8–10% SDS polyacrylamide gels. Proteins were transferred onto a polyvinylidene fluoride membrane using a BioRad gel blotting apparatus. Membranes were incubated with a primary antibody against A20 (ab13597, Abcam), DDI2 (sc-514004, Santacruz), and lysozyme (15013-1-AP, Proteintech) overnight at 4 °C and with a peroxidase-conjugated secondary antibody for 60 min. To confirm equivalent sample loading, β-actin was used as an internal control.

### N6-methyladenosine (m6A) Nanopore sequencing

The experimental process was performed in accordance with the standard protocol provided by Oxford Nanopore Technologies (ONT), including sample quality detection, library construction, library quality detection and library sequencing [14]. In brief, high-quality total RNA was extracted, and Nanodrop and Agilent 2100 were used for concentration, purity and integrity checks. The oligo (dT) magnetic beads enriched mRNA, and then constructed library (SQK-RNA002 direct RNA sequencing kit), including magnetic bead purification, Qubit library quantification, and MinION sequencing. ONT sequencing data were analyzed by BENAGEN, and the Q value was set to 7 to obtain pass reads and base calling was performed using Guppy v3.4.5. The MINES (m6A Identification using Nanopore Sequencing) software was used to detect m6A sites on RNA by random forest models, each corresponding to a DRACH motif including AGACT, GGACA, GGACC, and GGACT. Differentially methylated sites were identified using SMART2 software. The screening criteria for differential sites are the specificity of methylation sites is ≥ 0.3 and *P* value is < 0.01. The genes where the differentially methylated sites are located are enriched using the R package ClusterProfilter.

### LC/MS analysis and ubiquitinated protein identifications

The ubiquitinated protein identifications were performed according to the previous studies [15, 16]. The lung tissues of newborn rats were used for protein extraction and in-solution digestion. The digest peptides of each sample were desalted on C18 Cartridges (Empore™ SPE Cartridges C18, Sigma), and lyophilized. The samples were reconstituted in 1.4 mL of precooled IAP Buffer, added pretreated Anti-K-ε-GG antibody beads (Cell Signal Technology), then incubated, centrifuged, and discarded the supernatant. Anti-K-ε-GG antibody beads were washed with 1 mL precooled IAP Buffer and precooled water. 40 μL 0.15% TFA was added to the washed beads, incubated for 10 min at room temperature, then added 0.15% TFA again, centrifuged, the supernatant was desalted by C18 STAGE Tips.

Liquid chromatography/mass spectrometry (LC/MS) analysis was performed on a Q Exactive mass spectrometer that was coupled to Easy nLC. The peptides were loaded onto a reverse phase trap column connected to the C18-reversed phase analytical column (Easy Column) in buffer A (0.1% Formic acid) and separated with a linear gradient of buffer B (84% acetonitrile and 0.1% Formic acid). The mass spectrometer was operated in positive ion mode. MS data was acquired using a data-dependent top10 method dynamically choosing the most abundant precursor ions from the survey scan (300–1800 m/z) for HCD (high-energy collisional dissociation) fragmentation. Survey scans were acquired at a resolution of 70,000 at m/z 200 and resolution for HCD spectra was set to 17,500 at m/z 200, and isolation width was 2 m/z. Normalized collision energy was 30 eV and the underfill ratio, which specifies the minimum percentage of the target value likely to be reached at maximum fill time, was defined as 0.1%. The instrument was run with peptide recognition mode enabled. Gene Ontology (GO) analysis was performed to characterize categorical gene function in the components identified.

### m6A-specific methylated RNA immunoprecipitation (MeRIP) and RT–PCR

Total RNA was isolated from lung tissues according to the RNeasy protocol (Axygen). The m6A-specifc MeRIP was performed according to the previous method [17]. In brief, purified mRNAs (5 μg) were digested by Dnase I (M0303, NEB) and then fragmented into around 200-nt fragments by incubation at 95 °C for 25 s in RNA Fragmentation Reagents (Ambion, AM8740), followed by standard ethanol precipitation and collection. Anti-m6A antibody (3 μg antibody) was incubated with 30 μL Dynabeads G (nvitrogen, 10003D) in IPP buffer (150 mM NaCl, 0.1% NP-40, 10 mM Tris–HCl, pH 7.4) for 2 h at room temperature. The mRNAs (100 μg) were incubated with the prepared antibody-bead mixture for 4 h at 4 °C. By washing three times, proteinase K and Super RNase Inhibitor were then incubated with Dynabeads. The Trizol-LS was added to the beads, mix well at room temperature for 5 min. The eluted RNA was extracted by Enol:Chloroform:Isoamylol (pH < 5.0, Solarbio life science, P1025) and then generated to cDNA using a reverse transcriptase kit (TAKARA) according to manufacturer’s protocols. The enrichment of m6A was quantified by real-time quantitative PCR (RT–PCR) and performed by the ABI Prism 7500 Instrument following the TAKARA protocol. Β-actin was used as an internal control. Primers for ET1, A20, and β-actin are as follows: forward: 5′-aagcagacaaagaactccgag-3′, reverse: 5′-cgctttcaactttgcaactcg-3′; forward: 5′-tttgtggagacgggactttg-3′, reverse: 5′-gaggccattttgaccaagttg-3′; forward: 5′-gccaaccgtgaaaagatg-3′, reverse: 5′-tgccagtggtacgaccag-3′, respectively.

### Statistical analysis

All the statistical analysis and graphics were performed with R statistical software packages (R version 4.0.3). The continuous variables between groups were compared by Student’s *t*-test or Mann–Whitney *U* test. A difference of *P* < 0.05 was considered to be statistically significant.

## Results

### Decreased A20 levels of lung tissues in IUGR rats

**Fig. 1:**
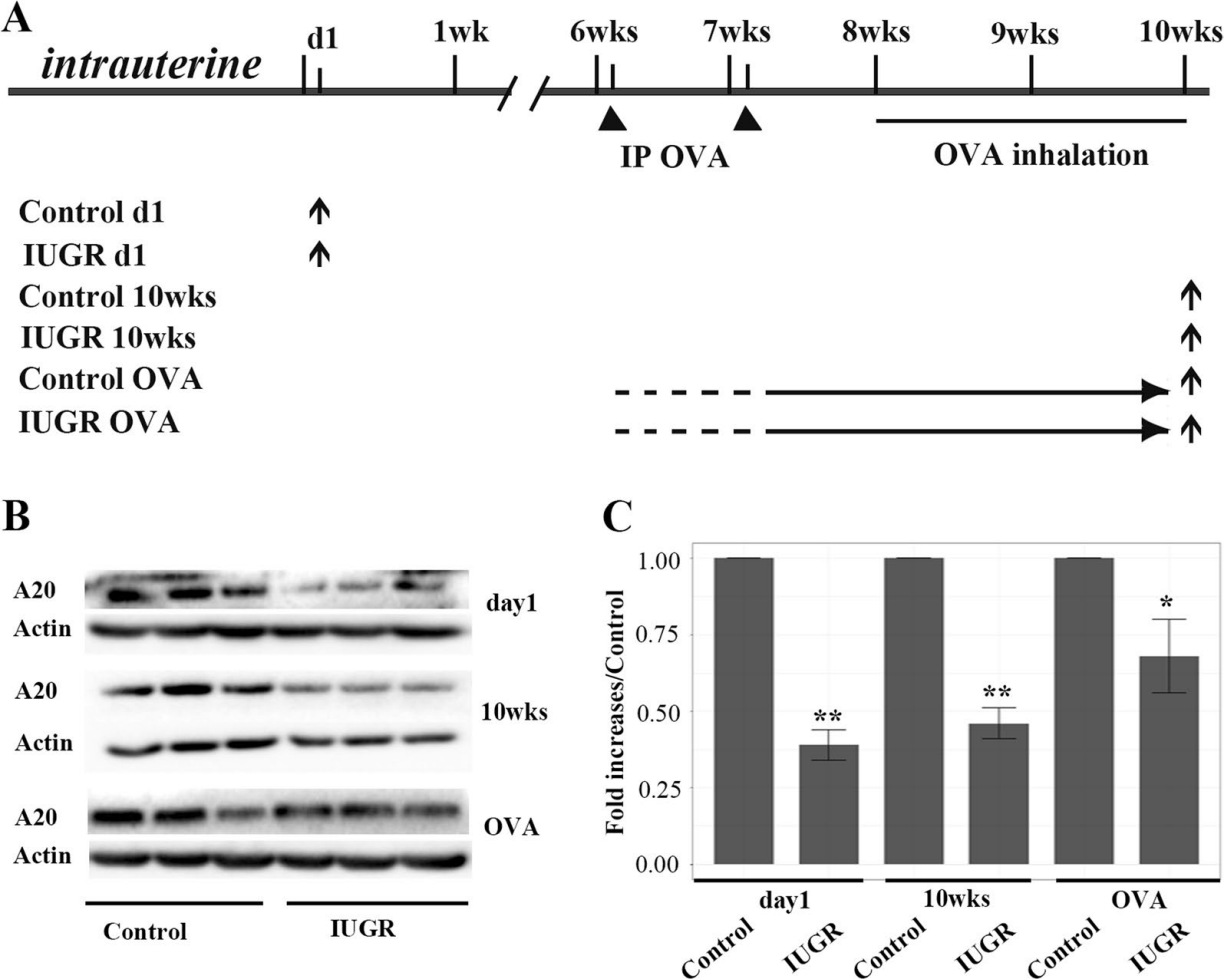
Establishment of IUGR OVA challenging model. **A** shows sampling age of different experimental group rats. Upward arrows represent sampling times, solid triangles represent the times of interventions, d1 represents 1 day after birth. **B** Western blot indicates A20 protein levels. β-actin protein expression served as an internal control and was used to normalize the protein band intensity. Noted decreased A20 levels in IUGR rats, from day1 to 10 weeks after birth, even OVA challenging (n = 3). **C** The bar graph showed increased folds of A20 in IUGR relative to control rats. IP = intraperitoneal injections; OVA = ovalbumin. *P < 0.05, **P < 0.01

The sampling age of different experimental group rats were seen in Fig. 1A. The A20 protein level of the IUGR d1 group was significantly lower that of the Control d1 group (*P* = 0.002, Fig. 1B and C). Compared with age-matched control group, A20 protein level of the IUGR 10wks group was significantly lower (*P* = 0.003, Fig. 1B and C). Furthermore, A20 expression of the IUGR OVA group was also significantly lower that of control OVA group, which yielded a significant difference (*P* = 0.012, Fig. 1B and C). These results indicate that IUGR can reduce the expression of A20 protein in lung tissue of newborn rats and continue until 10 weeks after birth. Although OVA challenge can increase the expression of A20 protein in lung tissue of IUGR rats (Fig. 1B), its level was still significantly lower than the control OVA group. Our previous study demonstrated that IUGR rats had a high sensitivity to OVA challenge later in life and presented with more severe asthma phenotype [11], which further suggesting that there exists a clear correlation between increased sensitivity to OVA and decreased A20 expression.

### Identification of differentially ubiquitinated proteins in lung tissue

**Fig. 2:**
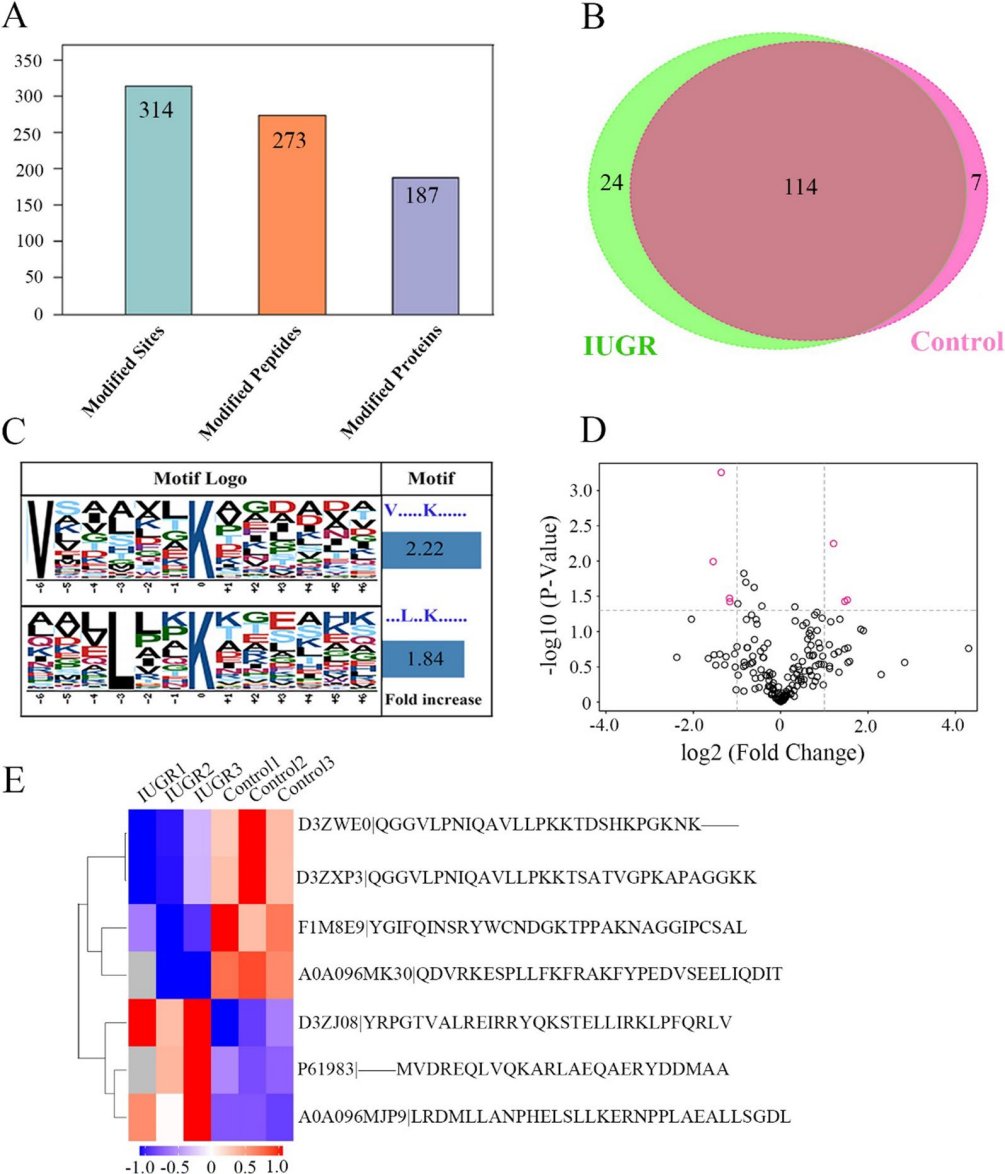
Identification of ubiquitinated proteins in lung tissues. **A** 314 ubiquinated sites, 273 ubiquinated peptides, and 187 ubiquinated proteins were identified in lung tissues. **B** The Veen diagram showed the overlap of identified ubiquitinated proteins between IUGR and control group. **C** The ubiquitination motifs and conservation of ubiquitination sites. The central K stands for the ubiquitinated protein. The size of each letter is related to the frequency of amino acid residues occurring at that position. **D** Volcano plot using two factors showing the difference in ubiquitinated peptides between the two groups of samples. The red dots represent significantly differentially expressed ubiquitinated peptides (fold change is greater than 2 and *P* value is less than 0.05). **E** Heatmap for differentially expressed ubiquitinated proteins between IUGR and control groups via label-free relative quantitative proteomics

Given the ubiquitin-modifying role of A20, ubiquitinated proteins of lung tissue were detected to further assess protein modification status of IUGR rats. A total of 314 ubiquinated sites, 273 ubiquinated peptides, and 187 ubiquinated proteins were identified in lung tissues using label-free quantitative proteomics technology among the IUGR and control newborn rats (Fig. 2A and B). To better elucidate regulation of ubiquitination in IUGR, ubiquitination motif analysis was carried out by examining the sequences of amino acid residues in the ubiquitination sites of the ubiquitinated peptides with Motif-X software [18]. Two significantly distinguished motifs were identified (Figs. 2C), including V-X(5)-K*-X(5) and X(3)-L-X(2)-K*-X(6), which refers to 44 and 40 unique ubiquitinated peptides, respectively (K* = the ubiquitinated lysine residue; X = any amino acid residue). In addition, the significant fold differences and clustering algorithm in ubiquitinated peptides between the two groups were seen in Fig. 2. Based on these differentially expressed ubiquitinated peptides, three increased and four decreased probable ubiquitinated proteins in IUGR d1 group were identified compared with control d1 group, including DNA damage-inducible 1 homolog 2 (DDI2), 14-3-3 protein gamma, histone H3, histone H2A (Hist2h2ab), histone H2A (H2afx), moesin, and lysozyme, respectively.

### Ubiquitinated protein status of IUGR probably continued into adulthood

**Fig. 3:**
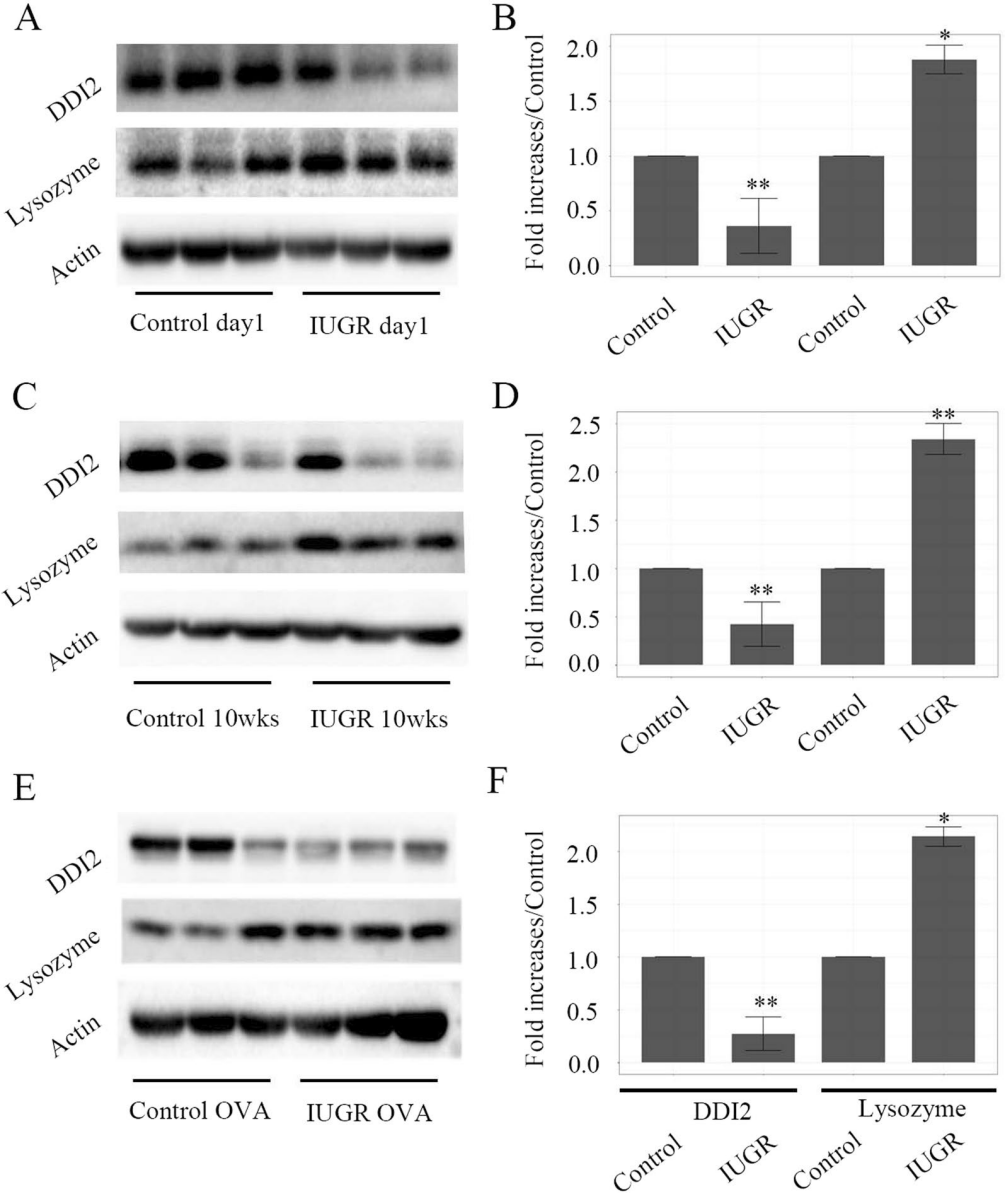
Expressions of lung tissue proteins were measured using Western blot analysis. Western blot indicates DDI2 and lysozyme expression levels of lung tissues in IUGR day1 (**A**), 10 weeks (**C**) and OVA challenging groups (**E**). The bar graph represents the relative protein levels (increased folds to control group, **B** for day1, **D** for 10wks, and **F** for OVA). β-actin protein expression served as an internal control and was used to normalize the protein band intensity. *P < 0.05, and **P < 0.01

We hypothesized that these differentially expressed ubiquitinated proteins in newborn IUGR rats might continue into adulthood, and still exist even under OVA challenge. Since the antibodies against ubiquitinated proteins were unavailable, we detected unmodified protein levels to assess relative ubiquitinated protein level. The decreased DDI2 and increased lysozyme expressions in IUGR d1, 10wks, and OVA groups were coincident with the increased ubiquitinated DDI and decreased ubiquitinated lysozyme levels, suggesting that this ubiquitination phenomenon is likely to continue from the newborn to adulthood (Fig. 3).

### Differentially m6A modifications of lung tissues in the newborn rats

**Fig. 4:**
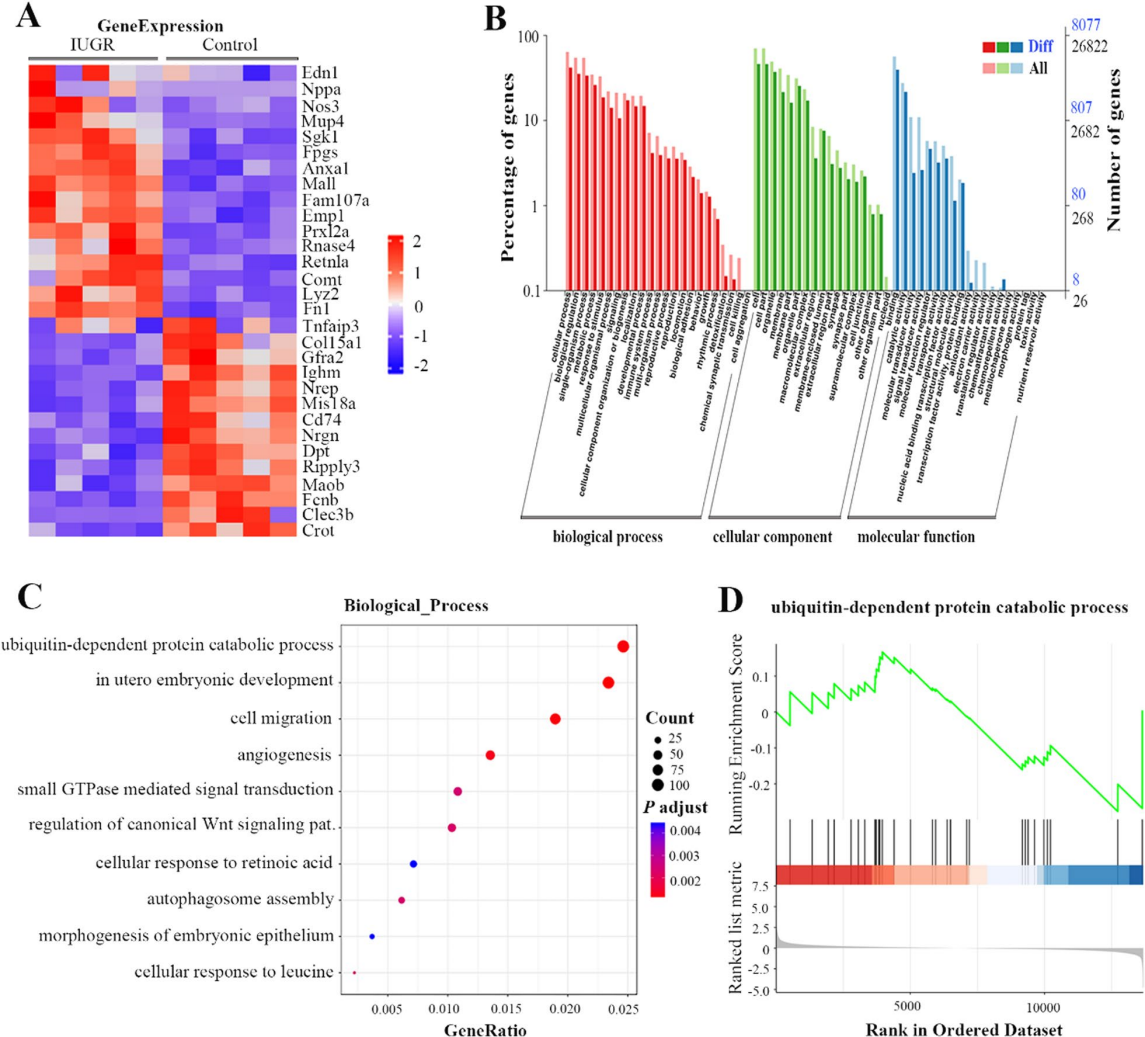
Transcript distributions of differential methylation loci (DML) in lung tissues. **A** Heatmap for differentially transcript expressions of DML between IUGR (n = 5) and control (n = 5) groups. **B** Gene ontology (GO) functional analysis on the transcripts of DML shows gene enrichment. The left side of the vertical axis is the percentage of transcript numbers, and the right side is the number of transcripts. **C** GeneRatio is the ratio of the transcripts of DML in the specific biologic process to the whole number of transcripts in all DMLs. Noted that the ubiquitin-dependent protein catabolic process had the highest abundance. **D** Gene set enrichment analysis (GSEA) of ubiquitin-dependent protein catabolic process. Running enrichment score (green curve) for the gene set. Grey line represents the location information of the sorted transcript set

The above results demonstrated that IUGR reduced A20 expression of lung tissue, and induced individuals to be highly sensitive to OVA challenge. Furthermore, the protein ubiquitination consistent with A20 changes in IUGR is likely to participate in the hyper-responsiveness to allergen challenge. Our previous study revealed that epigenetic mechanisms (histone acetylation) might be closely associated with the development of asthma following IUGR [11]. Therefore, we speculate that epigenetics such as N6-methyladenosine (m6A) of mRNA might be involved in A20 gene regulation. The heatmap of Fig. 4A showed representative transcripts where differential methylation loci (DML) located in lung tissues between IUGR d1 and control d1 groups, indicating increased ET1 (Edn1) expression and decreased A20 (Tnfaip3) expression in newborn IUGR rats. The transcript profiling of DML involved in all the biological functions including biological process, cellular components and molecular function (Fig. 4B). Notably in the detected transcripts on biological process, ubiquitin-dependent protein catabolic process had a greater abundance (Fig. 4C and D), further indicating that there might be an abnormal ubiquitinated pathway in IUGR rats.

**Fig. 5:**
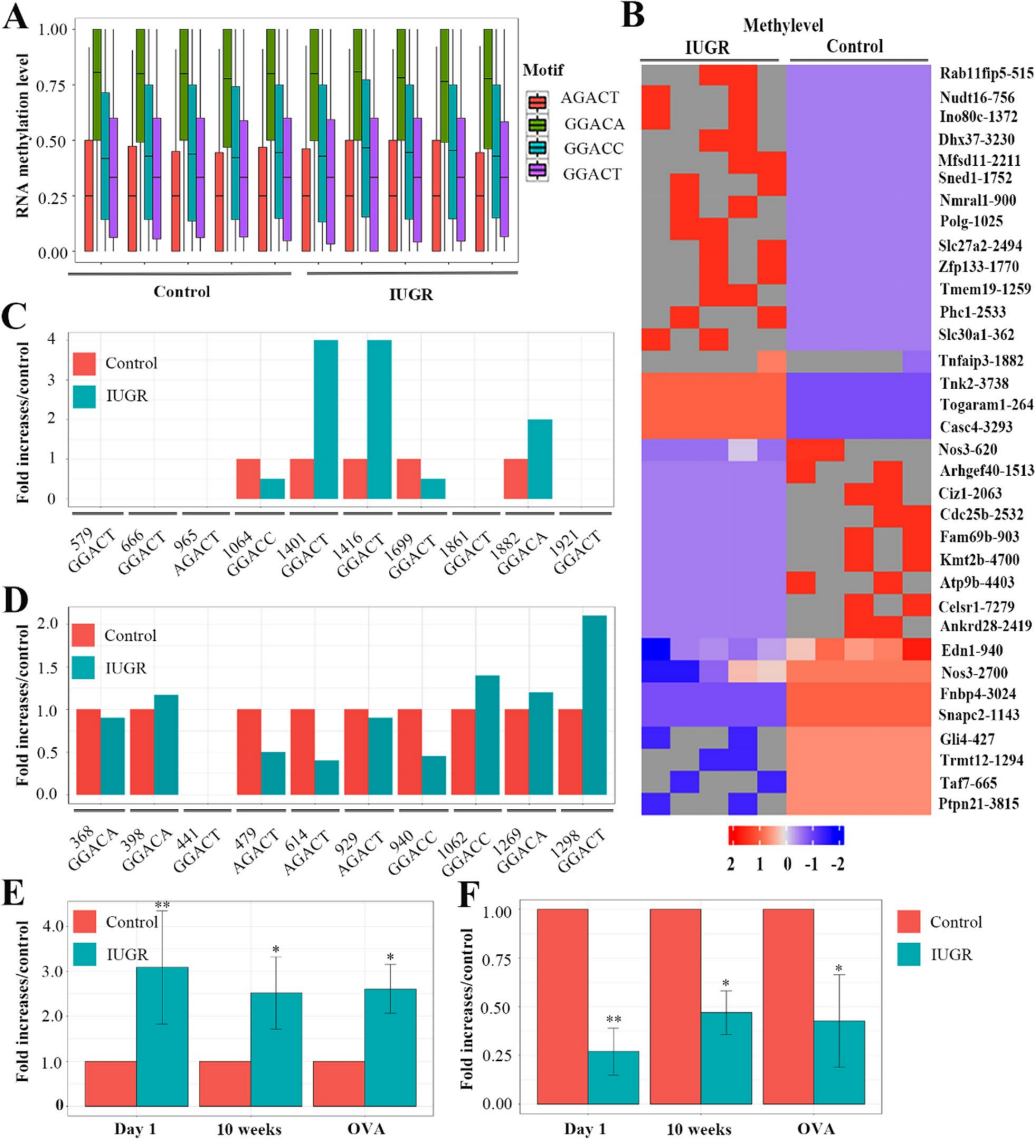
m6A modifications of lung tissues. **A** The diagram showed RNA methylation levels of four motifs. Noted that the motif of GGACA had the highest abundance. **B** The heatmap for the specific mRNA sites of m6A modifications in lung tissues between IUGR d1 and control d1 groups. **C** The relative m6A modification levels of ten specific sites of A20 mRNA in IUGR rats. **D** The relative m6A modification levels of ten specific sites of endothelin-1 mRNA in IUGR rats. **E** The bar graph shows that m6A modifications of A20 mRNA in newborn IUGR rats was significantly higher that control group, and persisted into 10 weeks after birth, even during OVA challenge. **F** The bar graph shows that m6A modifications of endothelin-1 mRNA in newborn IUGR rats was significantly lower that control group, and persisted into 10 weeks after birth, even during OVA challenge. *P < 0.05, **P < 0.01

In the detected RNA methylations, m6A abundance of the motif GGACA was highest, followed by GGACC, GGACT, and AGACT (Fig. 5A). The specific mRNA sites of m6A modifications in lung tissues between IUGR d1 and control d1 groups were shown in the form of a heatmap (Fig. 5B). We found that IUGR had a higher abundance of m6A modification for A20 mRNA, and a lower abundance of m6A modification for ET1 mRNA compared with control group. Further analysis showed that the sites 1401, 1416, and 1882 of A20 mRNA had higher m6A modifications in IUGR rats. However, no m6A modification was detected in the sites 579, 666, 965, 1861, and 1921 between the two groups (Fig. 5C). In contrast, most of sites for ET1 mRNA showed a lower abundance of m6A modifications in IUGR rats (Fig. 5D).

### Increased m6A modification of A20 gene in lung tissues of IUGR rats

To further investigate whether the m6A modification can maintain from the newborn to adulthood, MeRIP was used to assess the abundance of m6A modifications of mRNA. We found that m6A modifications of A20 mRNA of lung tissue in newborn IUGR rats were significantly higher that control group, and persisted into 10 weeks after birth, even during OVA challenge (Fig. 5E). This result indicated that decreased A20 protein levels in IUGR rats might be strongly associated with increased m6A modifications of A20 mRNA. Our previous study showed that increased ET1 expressions in IUGR rats were associated with histone acetylation of ET1 gene promotor. Here, we also demonstrated that increased ET1 expressions in IUGR rats might be correlated with its reduced m6A modification (Fig. 5F). These results further suggested an important role of m6A in asthma development following IUGR.

## Discussion

A large number of studies have recently confirmed the important role of A20 in the pathogenesis of asthma [5, 7]. A study by Riffo-Vasquez showed that A20 expression in asthmatic human lung was significantly lower in comparison to healthy subjects, inversely correlated with eosinophil migration, supporting the hypothesis that A20 has a protective effect on asthma and allergy [19]. Additionally, A20 gene and protein expression was consistently decreased, whereas proinflammatory Toll-like receptor expression was increased in urban asthmatic patients, reflecting their increased inflammatory status. Newborns with asthma at school age had reduced A20 expression at birth, suggesting A20 as a possible biomarker predicting subsequent asthma [10]. A20 can also regulate T helper 2-mediated eosinophilic and neutrophilic airway inflammation via pulmonary conventional type 1 Langerin-expressing dendritic cells and myeloid cells, respectively [9, 20].

Further research indicated that chronic exposure to low-dose endotoxin or farm dust protects mice from developing house dust mite-induced asthma; loss of A20 in lung epithelium abolished the protective effect, which suggesting the important role of A20 in the farming environment protects from allergy [7]. Furthermore, a study about the protective effects of tuberculosis against allergic diseases has unveiled that the up-regulated A20 contributed to maintaining a long-lasting anti-inflammatory and immunomodulatory activity induced by *Mycobacteria tuberculosis* chaperonin 60.1 [21]. Additionally, A20 could also alleviate allergic asthma in mice by promoting the production of Treg cells in the form of poly(lactic-co-glycolic) acid-OVA + A20 (PLGA-OVA + A20) nanovaccine [22]. Similar to previous studies, our study also demonstrated that IUGR reduced the A20 expression of lung tissue and persist into adulthood, and induced IUGR individuals to be highly sensitive to OVA stimuli, further confirming the protective role of A 20 in the development of asthma following environmental changes in early life.

A20 can regulate the ubiquitinated process participating in various inflammatory reaction in addition to suppressing NK-kB activity. Ubiquitination belongs to the post-translational modification, which decides the fate of various proteins in the cells, by either directing them towards proteasomal degradation or involving in cell signaling pathways. E3 ubiquitin ligases act as one of the most important enzymes affecting the process of ubiquitination [23]. The ubiquitin–proteasome system can degrade most intracellular proteins, including membrane-surface receptors. The proteolytic processing of a surface receptor by ubiquitin-mediated degradation profoundly attenuated pulmonary inflammation induced by endotoxin-containing pathogens [24]. Recently, more and more studies have also identified the ubiquitination as a central process in the pathogenesis and development of asthma [23, 25]. Ubiquitination also indirectly participated in the pathogenesis of asthma through regulating functions of macrophage and dendritic cells [26, 27].

To further explore the role of ubiquitination associated with A20, we performed the ubiquitinomics on lung tissues of newborn IUGR rats. We found that IUGR newborn rats had increased ubiquitination levels of DDI2, protein gamma and histone H3, and reduced ubiquitination levels of histone H2A, moesin, and lysozyme relative to normal newborn rats. Furthermore, this trend can continue into adulthood. As the antibodies against ubiquitinated proteins are unavailable here, we did not directly assess ubiquitination levels of proteins. Given the fact that the ubiquitinated modifications are likely to cause increased protein degradation and subsequently reduced protein levels, we speculate that ubiquitinated proteins might result in decreased detectable protein levels. Therefore, we utilize Western Blot method to detect unmodified proteins to indirectly reflect the level of ubiquitination. Our results showed that the decreased unmodified DDI2 levels in IUGR lung tissues were consistent with increased ubiquitinated DDI2, suggesting a differential ubiquitination between IUGR and control rats. However, their potential role in IUGR needs further investigation.

In the present study, we noted that the decreased A20 expression and differential ubiquitination of lung in IUGR might be involved in an increased risk of asthma later in life. To investigate whether reduced A20 levels of lung in IUGR were associated with m6A modifications, m6A nanopore sequencing was performed. The m6A is a chemical modification present in multiple RNA species, being most abundant in mRNAs. Studies on enzymes or factors that catalyze, recognize, and remove m6A have revealed its comprehensive roles in almost every aspect of mRNA metabolism, as well as in a variety of physiological processes. Accumulated studies reveal that m6A modification can either enhance or attenuate mRNA stability dependent on the type of m6A reader protein [28]. DNA hypomethylation and histone hyperacetylation at the A20 promoter can activate A20 expression in Zn-supplemented hens [29]. Inverse correlations between the A20 mRNA and DNA methylation levels in its intron indicated that increased A20 expression seems to be regulated by DNA demethylation in GC samples [30]. The downregulation of A20 in CD4 + T cells from systemic lupus erythematosus was closely associated with demethylation of histone H3K4, which led to a decreased amount of H3K4me3 in the promoter of the A20 gene [31]. These results revealed that the expression of A20 gene could be regulated by epigenetics, including histone modifications and DNA methylation. However, m6A modification of A20 gene was not fully understood. The present result demonstrated that the increased m6A modification of A20 mRNA was closely associated with decreased A20 proteins. This also further indicated the expression of A20 gene was involved in m6A modification. Moreover, the m6A modification of A20 mRNA would contribute to mRNA degradation and reduced A20 protein levels.

In conclusion, IUGR can reduce the expression of A20 protein in lung tissue of newborn rats and continue until 10 weeks after birth. The decrease A20 expression induces IUGR individuals to be hyper-sensitive to OVA challenge. The phenomenon was closely associated with ubiquitination of A20 proteins and m6A modifications of A20 mRNA. These findings indicate A20 as a key regulator during asthma development following IUGR, providing further insight into the prevention of asthma induced by environmental factors.

## Data Availability

The datasets used and/or analyzed during the current study are available from the corresponding author on reasonable request.
